# Invasive Partial Hydatidiform Mole Presenting As Cesarean Scar Pregnancy: A Case Report

**DOI:** 10.7759/cureus.104027

**Published:** 2026-02-21

**Authors:** Rubandra Kumaar Kalimuthu, Siti Zawiah Omar, Yin Ling Woo, Vallikannu Narayanan, Anushya Vijayananthan

**Affiliations:** 1 Department of Obstetrics and Gynecology, University Malaya Medical Centre, Kuala Lumpur, MYS; 2 Department of Obstetrics and Gynecology, University Malaya, Kuala Lumpur, MYS; 3 Department of Biomedical Imaging, University Malaya, Kuala Lumpur, MYS

**Keywords:** case report, cesarean scar pregnancy, ectopic pregnancy, invasive hydatidiform mole, partial hydatidiform mole

## Abstract

Invasive hydatidiform mole is a rare subtype of gestational trophoblastic disease characterized by myometrial invasion and the potential for significant morbidity. Cesarean scar pregnancy (CSP) is an uncommon type of ectopic pregnancy with increasing incidence due to the rise in cesarean delivery rates. The coexistence of an invasive hydatidiform mole implanted in a cesarean scar is exceptionally rare and poses significant diagnostic and therapeutic challenges. This is a case of a 32-year-old Malay woman with a prior history of cesarean section who presented with per-vaginal bleeding in early pregnancy. Assessment revealed a viable gestational sac with vesicles within, suggesting that a partial hydatidiform mole had implanted at the cesarean scar site. Serum beta-human chorionic gonadotropin (β-hCG) levels were markedly elevated (>200,000 IU/L). MRI of the pelvis was reported as suggestive of partial invasive molar pregnancy with serosal breach through the cesarean scar and bladder invasion, along with a fetus seen, and proceeded with six cycles of methotrexate and folinic acid regimen. Serum β-hCG levels reduced accordingly (368 IU/L pre-sixth cycle and prior surgery). Proceeded with total abdominal hysterectomy, bilateral salpingectomy, and histopathological examination confirmed the diagnosis of an invasive hydatidiform mole arising from a CSP. The patient was managed with a multidisciplinary approach with close β-hCG surveillance. Clinical and biochemical remission was achieved. Invasive hydatidiform mole presenting as a CSP is extremely rare. Early recognition through imaging, β-hCG monitoring, and histopathology is crucial to prevent life-threatening complications. Prompt multidisciplinary management and careful follow-up are essential for favorable outcomes.

## Introduction

Molar pregnancy, or hydatidiform mole, is a form of gestational trophoblastic disease (GTD), which encompasses two forms of abnormal pregnancy: complete and partial hydatidiform moles. Malignant potential is greater for complete moles (15%) compared to partial moles (1%) [1]. The malignant spectrum of (GTD), collectively termed gestational trophoblastic neoplasia (GTN), includes invasive mole, choriocarcinoma, placental site trophoblastic tumor, and epithelioid trophoblastic tumor. GTN may arise following any type of pregnancy, including ectopic pregnancy, spontaneous abortion, or term pregnancy [1]. Invasive mole occurs when molar villi invade the myometrium or adjacent structures and is more commonly associated with complete moles. Because of its rarity, the precise incidence of molar pregnancy is difficult to ascertain, though global estimates range from 0.6 to 8 per 1,000 pregnancies [2].

Cesarean scar pregnancy (CSP) represents a rare ectopic pregnancy characterized by implantation at the myometrial defect of a prior uterine incision [3]. A potentially life-threatening variant of ectopic pregnancy. Previously regarded as exceedingly rare, CSP now has an estimated incidence of one in 1,500 cesarean deliveries, largely due to the marked increase in cesarean section rates worldwide over the last two decades [3].

The concomitant occurrence of invasive partial hydatidiform mole and CSP is extremely rare, with only a limited number of cases reported in the literature. Hence, diagnosing and managing such patients can be challenging. This case report highlights the importance of beta-human chorionic gonadotropin (β-hCG) and imaging in diagnosing such a patient with an invasive partial mole implanted at CSP. She was managed with chemotherapy, followed by a hysterectomy, conserving bilateral ovaries.

## Case presentation

**Figure 1:**
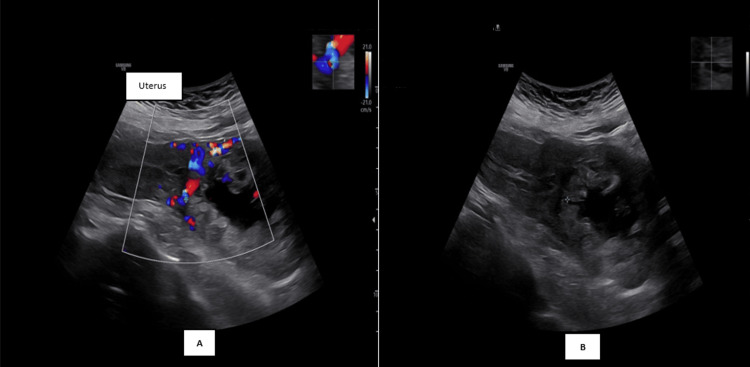
(A) Transabdominal ultrasound Doppler image showing an abnormal, highly vascularized sac located on the lower pole of the uterus. (B) Transabdominal ultrasound image without Doppler

**Figure 2:**
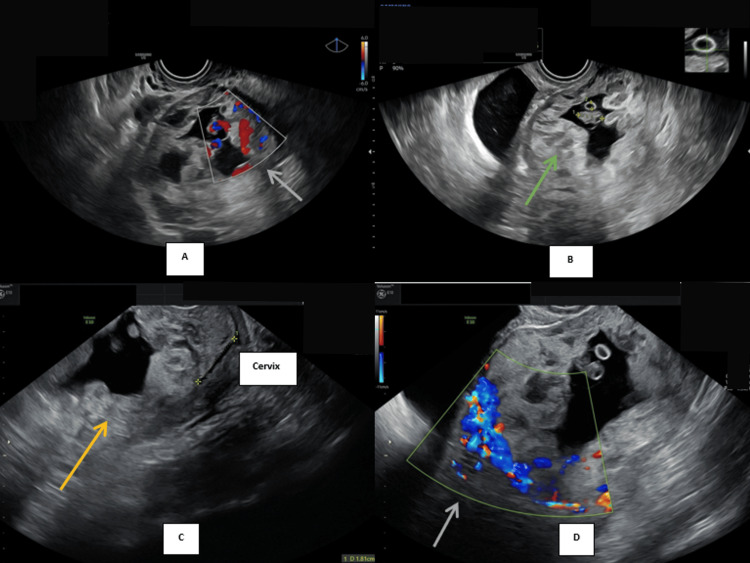
(A&D) Transvaginal ultrasound Doppler image (grey arrows) showing vascularized vesicles within the gestation sac. (B) Sac containing vesicles (green arrow) seen with fetal echo at the lower pole of the uterus. (C) Location of the sac (yellow arrow) in relation to the cervix

**Figure 3:**
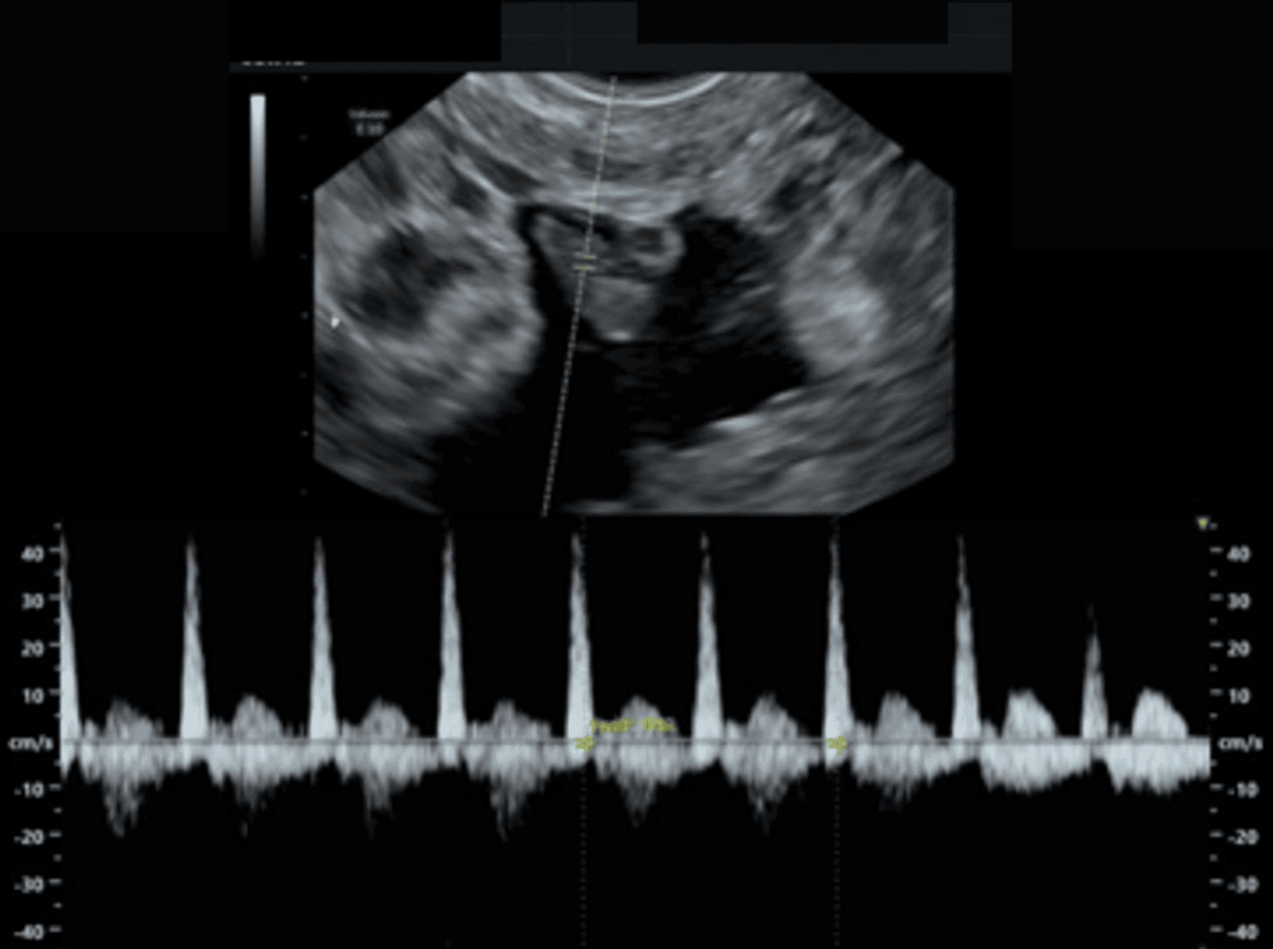
Transvaginal ultrasound fetal echo

**Figure 4:**
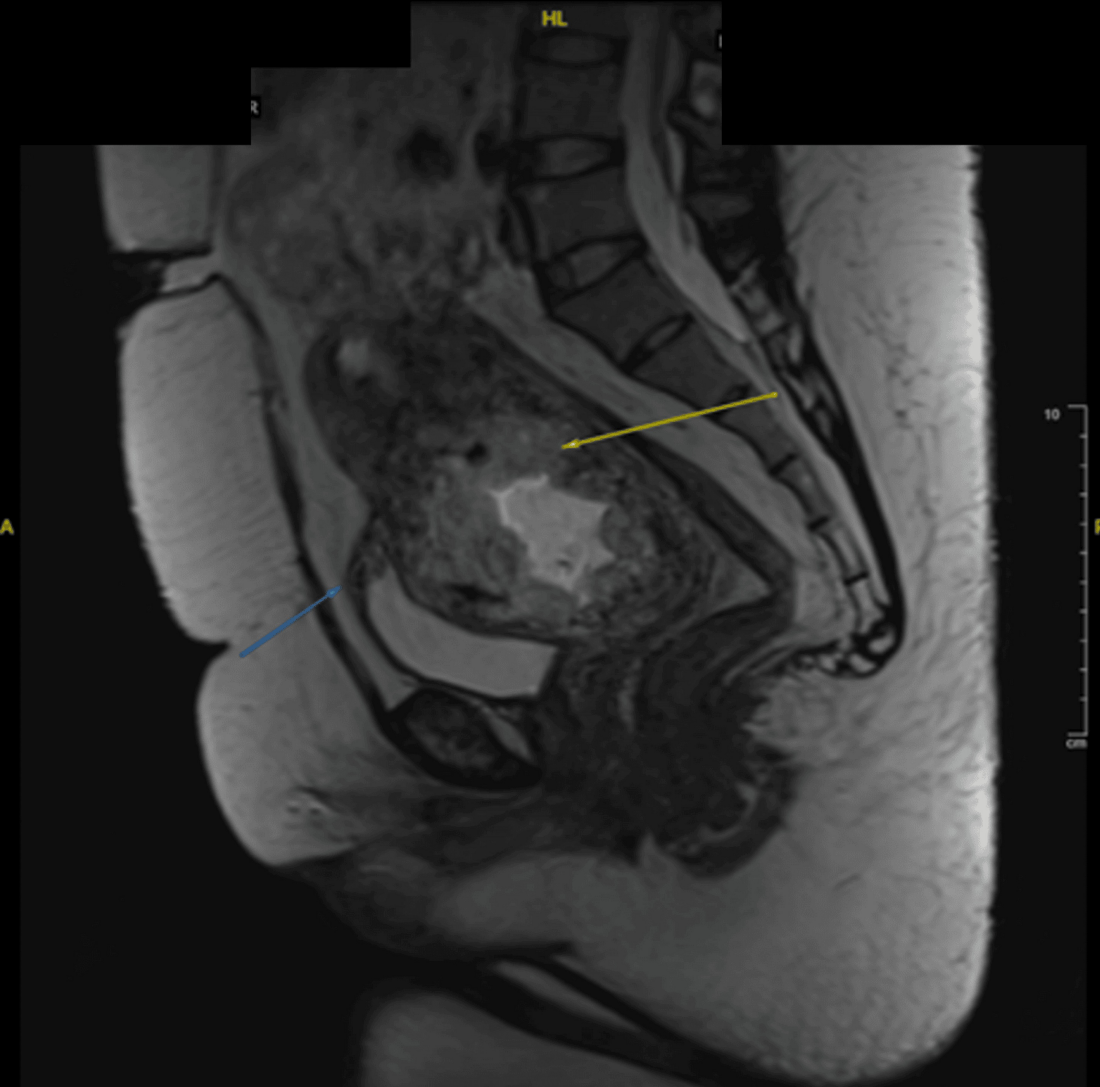
MRI upon diagnosis showing a likely molar pregnancy with serosal breach through the cesarean scar (yellow arrow) and urinary bladder invasion (blue arrow)

A 32-year-old Malay woman, G3P2 (one term vaginal delivery and one lower segment cesarean section), presented with early trimester per-vaginal bleeding. Assessment at an eight-week period of amenorrhea with ultrasound, as shown in Figure 1B, Figure 2B, and Figure 3, noted a gestation sac with a viable fetus and also vesicles seen implanted on the lower part of the uterus. The myometrium and the uterovesical interface appeared very thin, and the area around the sac was vascularized, as shown in Figure 1A, Figure 2A, and Figure 2D. The β-hCG level was >200,000 IU/L. Findings raised the suspicion of a partial hydatidiform mole CSP. Proceeded with MRI of the pelvis as demonstrated by Figure 4, which was reported as suggestive of molar pregnancy with serosal breach through the cesarean scar and urinary bladder invasion. The patient agreed to the termination of pregnancy after counseling.

Unable to perform intracardiac potassium chloride injection due to the presence of tortuous vessels around the sac (Figure 2A). A methotrexate regimen (50 mg/m² with folinic acid) was given for six cycles, during which a significant reduction in β-hCG was observed, from >200,000 IU/L to 368 IU/L prior to surgery.

**Figure 5:**
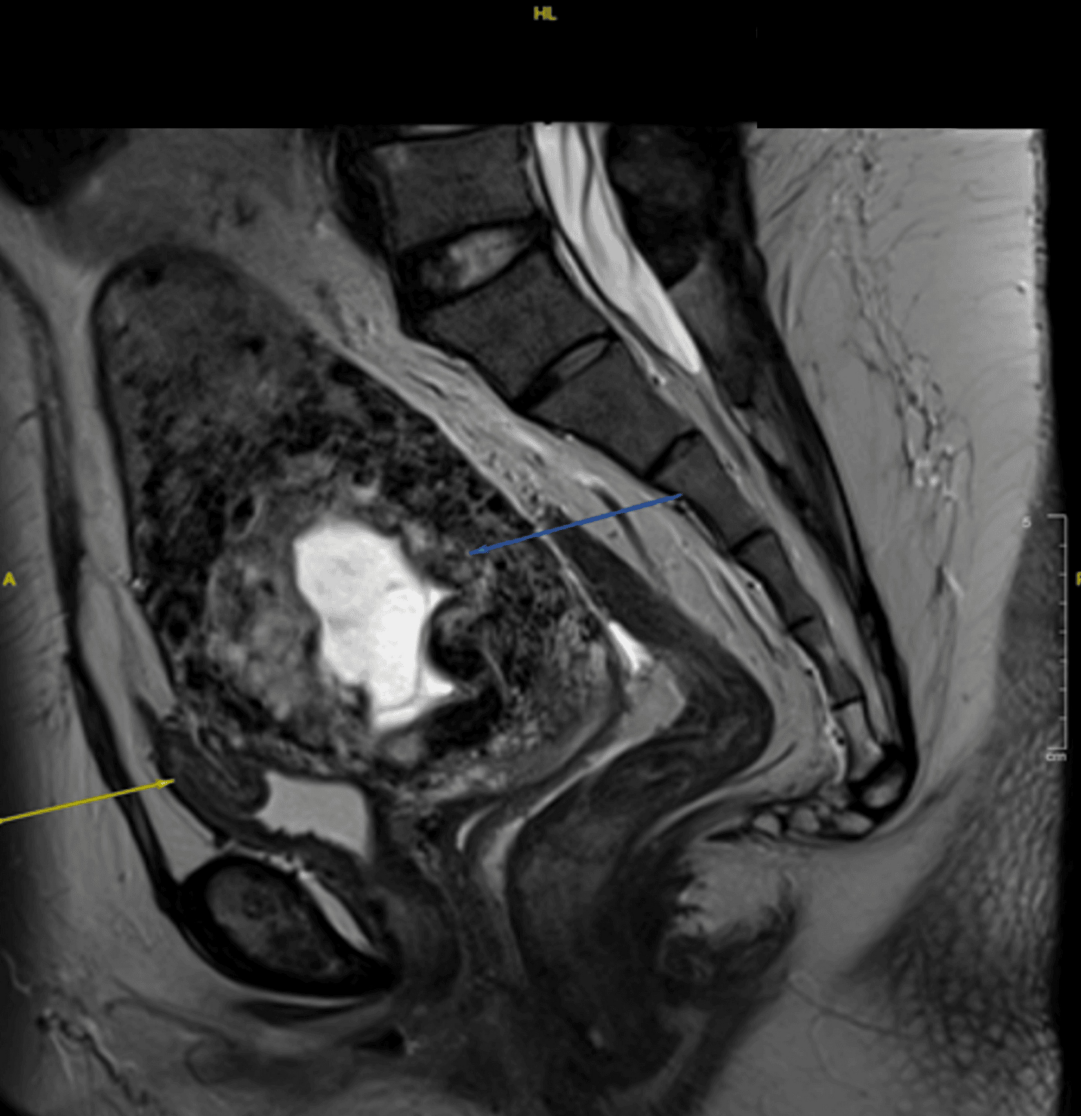
MRI upon completion of chemotherapy showed a stable partially invasive molar pregnancy breaching through the cesarean scar (blue arrow) with urinary bladder invasion (yellow arrow)

**Figure 6:**
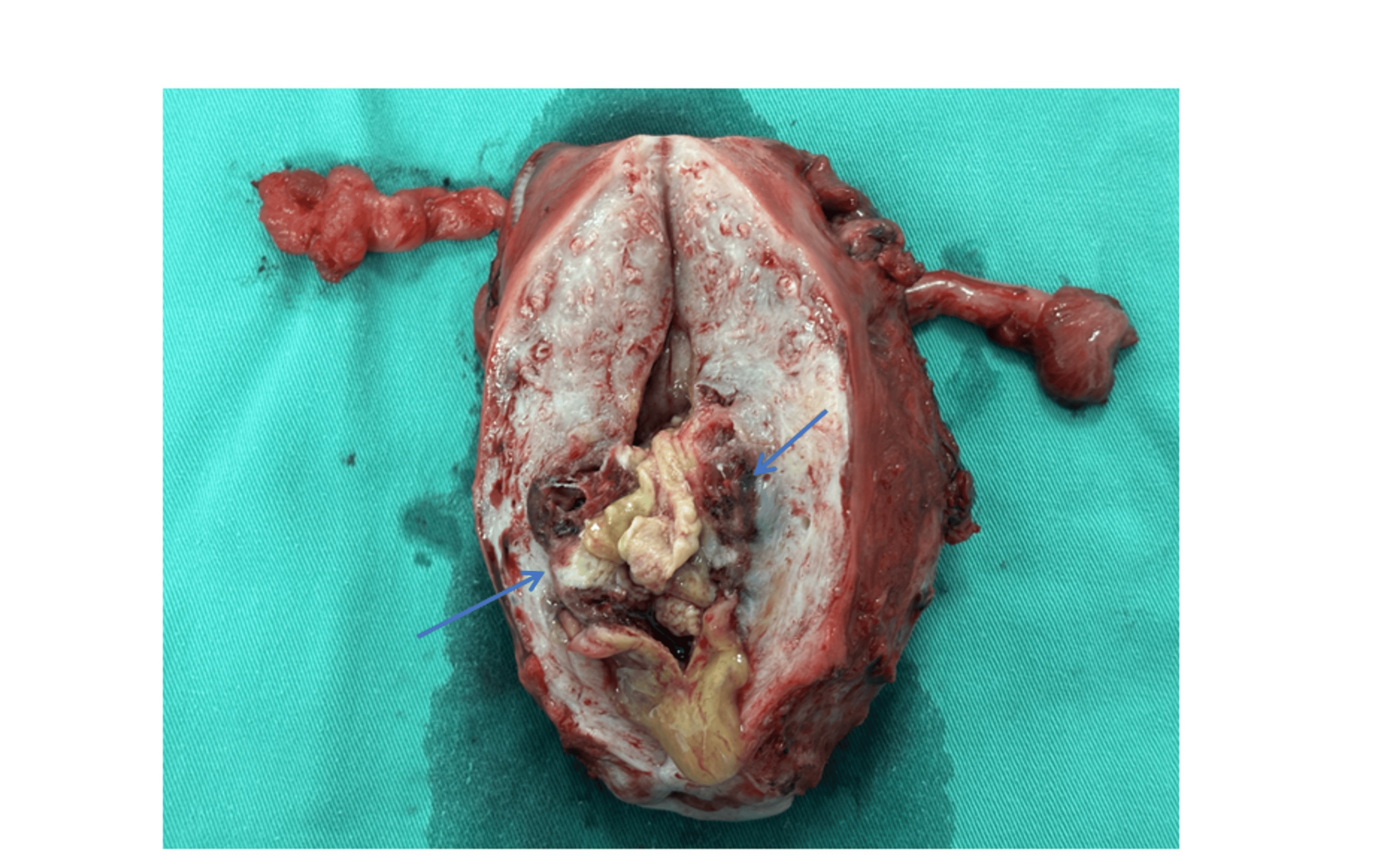
Uterine specimen showing mass at the previous cesarean scar site (blue arrows)

MRI post-chemotherapy/prior surgery showed a stable partially invasive molar pregnancy breaching through the cesarean scar with urinary bladder invasion, along with a deformed fetus within the uterine cavity (Figure 5). Intraoperatively, there was no obvious uterine serosa or bladder infiltration. Hysterectomy and bilateral salpingectomy were done, preserving the bilateral ovaries. An incision was made on the uterus; a mass was seen intrauterine, invading into the anterior uterine wall, involving more than half of the myometrium lining, especially at the previous scar site (Figure 6). These findings indicate a clear reduction in disease extent following chemotherapy.

As clinically predicted, histopathological examination revealed an invasive hydatidiform mole. This emphasizes the importance of clinico-radiological correlation for such a case, where obtaining a tissue biopsy prior to was not possible. Moreover, the use of chemotherapy prior to surgical intervention helped reduce the morbidity.

Histopathological examination reported as invasive hydatidiform mole with massive areas of coagulative and fibrinoid necrosis (80-90% of tumor bulk), with residual viable hydropic chorionic villi lined by proliferating cytotrophoblasts and syncytiotrophoblasts penetrating deep into the myometrial wall.

## Discussion

The development of hydatidiform moles has been linked to cytogenetic abnormalities, viral infections, and endocrine disorders [4]. A uterus larger than dates, abnormally elevated β-hCG, vomiting, theca-lutein cysts, and hypertension in early pregnancy are the clinical manifestations associated with this condition [4]. Invasive moles typically follow complete molar pregnancies; development from partial moles is rare and accounts for a small proportion of GTN cases. The diagnosis is often established based on elevated or rising serum β-hCG levels, imaging findings, and histopathological confirmation. An invasive mole is defined as a tumor in which molar villi penetrate the myometrium [4].

CSP, on the other hand, represents a unique implantation site that may predispose to abnormal trophoblastic invasion due to defective decidualization and myometrial fibrosis at the scar site. The exact pathophysiology leading to the development of ectopic cesarean scar pregnancies has not yet been fully elucidated [5]. However, these factors may facilitate deeper trophoblastic penetration, increasing the risk of invasive disease. In the present case, implantation of a partial mole within the cesarean scar likely contributed to early myometrium invasion. Ultrasonography with color Doppler is the primary diagnostic modality [6]. Rotas et al. reported that USG has a sensitivity of 84.6% for detecting CSP [7]. The presence of a gestational sac within the anterior lower uterine segment at the level of the scar, heightened peritrophoblastic vascularity, and myometrial thinning between the sac and bladder are suggestive features [8].

However, differentiating molar CSP from non-molar CSP can be very challenging. Markedly elevated or disproportionate β-hCG levels relative to gestational age may raise suspicion of GTD. Still, partial moles often have lower β-hCG levels than complete moles, further complicating diagnosis. Elevated β-hCG, the presence of fetal echo, and molar invasion into the bladder through the previous cesarean scar were the key points of this case. As it was not feasible to obtain tissue prior to commencing treatment, diagnostic decisions were guided by radiological findings in conjunction with clinical and biochemical features.

Management of invasive mole in a cesarean scar is extremely rare and lacks standardized protocols. Therefore, it must be individualized, taking into account hemodynamic stability, desire for future fertility, extent of invasion, and β-hCG trends. Options include surgical evacuation, hysterectomy, uterine artery embolization, and chemotherapy [9]. GTN is highly sensitive to chemotherapy, and single-agent regimens are often effective in low-risk disease [10].

**Figure 7:**
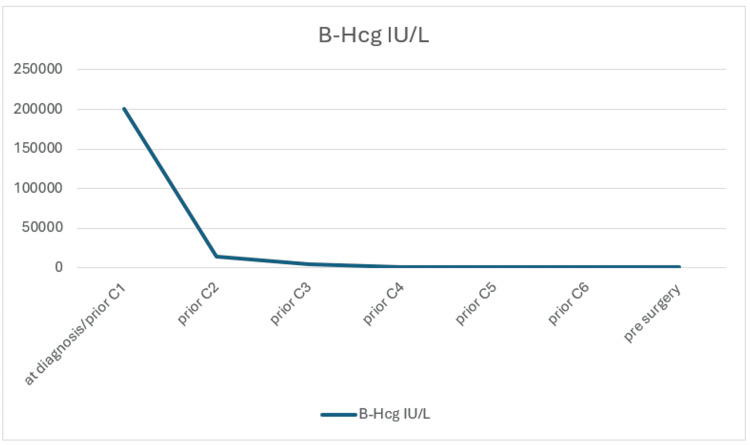
β-hCG trend β-hCG: beta-human chorionic gonadotropin

The patient's only risk factor is her ethnicity, being of Asian ethnicity. Due to the high risk of bleeding and being hemodynamically stable, she was started on chemotherapy (methotrexate and folinic acid). She received six cycles, and serum β-hCG levels decreased from >200,000 IU/L to 407 IU/L prior to surgery (Figure 7). A decision was made for hysterectomy and bilateral salpingectomy with ovarian preservation due to the extent of invasion. Intraoperatively, no obvious invasion was seen into the bladder, but tortuous vessels were seen in the area of the uterovaginal peritoneal fold. This discrepancy between MRI and intraoperative findings could be due to chemotherapy response. Other possible MRI finding explanations could be due to inflammatory changes or vascular congestion. Surgery was otherwise straightforward. Serum β-hCG two weeks post-surgery normalized to <2 IU/L, subsequently followed up for six months.

## Conclusions

The case emphasizes the importance of considering GTD in patients with atypical CSP features, particularly when abnormal β-hCG levels or unusual imaging findings are present. Early diagnosis and appropriate multidisciplinary management can significantly reduce morbidity and preserve reproductive potential. This underscores the indispensable role of radiological assessment and input in this case.
